# Family cultural capital and academic achievement: the mediating roles of habitus and field

**DOI:** 10.3389/fpsyg.2025.1745371

**Published:** 2026-01-15

**Authors:** Minglei Jin, Dirk Christiaan Gootjes, Hangfei Zhao, Ya Gu

**Affiliations:** 1 Department of General Education, Tourism College of Zhejiang, Hangzhou, China; 2 Jing Hengyi School of Education, Hangzhou Normal University, Hangzhou, China; 3 Endicott College, Woosong University, Daejeon, Republic of Korea; 4 Institute of High-Quality Education Development, Zhejiang Normal University, Jinhua, China

**Keywords:** academic performance, Chinese college students, educational equity, family cultural capital, theory of practice

## Abstract

**Introduction:**

Based on Bourdieu’s Theory of Practice, this study examines the mechanisms through which family cultural capital influences academic achievement among Chinese college students. While existing research has often fragmented the interplay between capital, habitus, and field, this study integrates these elements into a coherent analytical framework: family cultural capital → habitus → field → academic performance. By addressing gaps in contextualized and integrated analyses, this research explores how familial resources are internalized and transformed within educational settings, with a focus on the unique socio-cultural context of Chinese higher education.

**Methods:**

A cross-sectional survey was conducted with 2,929 students from 12 Chinese higher education institutions, selected through stratified and convenience sampling. Data were collected via a five-point Likert-scale questionnaire measuring family cultural capital, individual habitus, school field, and self-reported academic performance. Structural equation modeling (SEM) and bootstrap mediation analysis were employed to test the hypothesized chain mediation model.

**Results:**

The direct effect of family cultural capital on academic performance was significantly negative (β = –0.226, *p* < 0.001). However, significant positive indirect effects emerged through the mediating roles of habitus (β = 0.368, 95% CI [0.284, 0.470]) and school field (β = 0.084, 95% CI [0.038, 0.146]). More importantly, the sequential mediating effect of family cultural capital on academic performance, sequentially through habitus and field, is statistically significant (β = 0.087, 95% CI [0.044, 0.140]).

**Discussion:**

The findings indicate that, within the Chinese context, family cultural capital does not directly enhance academic performance. Instead, its benefits are realized indirectly through the development of adaptive habitus and alignment with school field dynamics. This aligns with Bourdieu’s emphasis on field-specific capital valuation and highlights the importance of contextual factors, such as Confucian-influenced parental expectations and institutional environments. The study underscores the need for educational policies that foster habitus development and field integration to mitigate inequities.

## 1 Introduction

The family, as the basic unit of social structural relations (Reiss, 1965), can provide material resources necessary for daily life and also realize functions such as emotional communication, relationship building, and individual socialization (Bernardes, 1999). No matter what changes occur in the current family organizational form and relationship structure, its social value is irreplaceable (Daly, 2005). Factors of family background, such as *family relationship and structure* (Lansford et al., 2001; Peterson, 2005), *family economy* (Lundberg and Pollak, 2007; Spencer and Komro, 2017), and *family social status* (Hogan and Kitagawa, 1985) exert a strong influence on the growth and development of teenagers. For the influence of family on the development of teenagers, Pierre Bourdieu’s *Theory of Cultural Capital* goes beyond the perspective of material economy, has strong explanatory power, and has been widely used in research (Davies and Rizk, 2018).

Family cultural capital’s value, the cultural property passed on by different family backgrounds, varies depending on the distance between family education and school education in different educational actions (Bourdieu and Passeron, 1977). Family cultural capital refers to the material cultural resources, cultural activities, cultural atmosphere, cultural space in the family environment, as well as the family cultural relations and structures beyond economic and social factors such as quality and literacy, academic certificates, and educational and parenting activities that can influence family members (Willekens and Lievens, 2014). Family cultural capital affects an individual’s psychology (Lehman et al., 2004), health (Abel, 2007), quality of life (Kim and Kim, 2009), and acquisition of social status (Georg, 2004). Family cultural capital even has an impact throughout the entire span of a person’s life (Georg, 2016).

However, existing research exhibits three limitations: First, most studies have fragmented the interactive ternary relationship among capital, habitus, and field in Bourdieu’s theory of practice, analyzing the effects of individual variables in isolation (Goldthorpe, 2007; Yang, 2014); second, some studies have focused solely on the direct effect or a single mediating effect of family cultural capital on academic achievement (Tan et al., 2019); and third, a few integrated studies have not incorporated the context of Chinese localization, resulting in limited theoretical explanatory power (Davies, 2024).

To address these research gaps, this study combines family cultural capital, habitus, and fields, using Bourdieu’s Theory of Practice system to construct the research framework, which can mitigate the limitation of the single-factor analysis of cultural capital. This novel framework could provide a clearer understanding of the complex influence mechanism of individual educational achievements and further deepen the application of cultural capital theory in the analysis of the influencing factors of college students’ academic performance. In addition, through the data analysis method of chain mediating effect, the theoretical system of educational sociology regarding the relationship between family and education could be expanded in the context of students’ academic achievement, enabling us to have a more comprehensive and in-depth understanding of the role of the mechanism through which family cultural capital influences educational outcomes.

This study utilizes Bourdieu’s Theory of Practice and takes individual habitus and the school field as mediators to examine the influence of family cultural capital on college students’ academic performance. The specific research objectives include: (1) studying the formation of college students’ academic achievements within Bourdieu’s Theory of Practice framework from the dimension of family cultural capital; (2) exploring the influence of family cultural capital on the academic performance of college students; and (3) determining whether habitus and fields mediate the relationship between family cultural capital and academic performance.

## 2 Theoretical framework and hypotheses development

### 2.1 Theory of practice

Bourdieu’s systematic exposition of the *Theory of Practice* first appeared in *Outline of a Theory of Practice* (1972). Through the analysis of field data in Algeria, Bourdieu put forward the basic viewpoints on the dialectical relationship between institutions and practices, individuals and society, epistemology and strategy, and symbols and social fields (Rasmussen, 1981). Since its conception, Bourdieu began to transcend structuralism and gradually formed his unique *Theory of Practice* (Acciaioli, 1981). In *An Invitation to Reflexive Sociology*, Bourdieu and Wacquant (1992), through reflection on the key concepts in his theoretical system, clarified the relationship among three factors (i.e., capital, habitus, and field), and combined them to form a tripartite *Theory of Practice*. Bourdieu’s ternary framework of capital, habitus, and field cut the “Gordian knot” by offering a more robust methodological toolbox for sociological research (Breiger, 2000). To demonstrate the characteristics of practical actions in different fields, Bourdieu (2015) synthesized the relationship logic between the three factors and the Theory *of Practice* using the formula:


[(Habitus)(Capital)]+Field=Practice

In the framework of Bourdieu’s Theory of Practice, it is important to properly address the issues of lag—the temporal mismatch between an individual’s cultural capital and the demands or values of a particular field—and adaptation, which refers to the process by which individuals adjust their dispositions and practices to align with the evolving norms and expectations of that field (Bathmaker, 2015).

Family cultural capital does not have a fixed value attribute. Its value is determined by the specific field and the different spaces and stages it is in. When taking family cultural capital as the analytical framework of the influence mechanism and function mode of social behavioral relations, it should be placed in a specific field (Lamont and Lareau, 1988). To better understand the operation mode, power system, and spatial position of the research object of the field, the structural relationship and operation principle of family cultural capital in this field should be analyzed. In addition, it is necessary to clarify the differences and connections between capital and habitus (Roksa and Robinson, 2016).

To better understand the relationship between family cultural capital and the field, it is important to move beyond abstract philosophical debates—such as those that focus solely on individual subjectivity (subject-centered approaches) or overarching structures (structure-centered approaches)—and instead focus on how individuals and structures interact in the distribution of power within the field. Likewise, it is important to not only overcome the constraints of structural philosophy, but also more clearly understand the practical significance of *the objective relationship of the field’s structure that acts on the agent and is manifested through the agent.*

This study focuses on the correlation among family cultural capital, habitus, field, and academic performance of college students. Therefore, it is assumed that using Bourdieu’s *Theory of Practice* as the research framework could reveal the correlation among these elements from multiple perspectives, presenting their dynamic changes and interaction processes, which could yield germane results that may inform further in-depth exploration of related issues in college student academic performance.

### 2.2 Hypotheses development

#### 2.2.1 Family cultural capital

In the sociology of education, family cultural capital has always been a research field of great concern (Davies and Rizk, 2018), since it was used by Bourdieu to study the cultural reproduction process of college students (Bourdieu and Passeron, 1979). Family cultural capital has become an important theory in the fields of equity in higher education and the development of college students. At present, most of the references to the expression of the concept of *cultural capital* in the literature come from *The Forms of Capital* (Bourdieu, 1986), in which Bourdieu divided cultural capital into objectified, embodied, and institutionalized forms. This classification has become the most important theoretical lens for researchers to define the category of cultural capital and has been widely used and disseminated (Davies and Rizk, 2018).

Within Pierre Bourdieu’s theoretical framework of cultural capital, the concepts of cultural capital and family cultural capital are frequently conflated. For the purpose of this study, *cultural capital* and *family cultural capital* are not coterminous but rather closely related constructs in Bourdieu’s cultural capital framework. In his works, *The Inheritors*, *Reproduction in Education, Society and Culture*, and *The State Nobility*, discussions of cultural capital consistently treat the family-background dimension as a core component. For example, in *The State Nobility*, a father’s occupational status, the size of the family library, and participation in highbrow cultural activities with parents are all treated as key indicators of cultural capital and mechanisms of social distinction (Bourdieu, 1996). When addressing embodied cultural capital, as mentioned in the book *The Forms of Capital:*

On the one hand, cultural capital in its objectified state depends above all on the cultural capital possessed by the family as a whole; on the other hand, the conditions for the rapid and easy accumulation of cultural capital are established from the outset by not delaying and not wasting time—an advantage that the offspring of families endowed with substantial cultural capital enjoy to the fullest (Bourdieu, 1986, p. 243).

Drawing on Bourdieu’s formulations, this study defines family cultural capital as the family’s stock of material cultural resources, cultural practices, cultural atmosphere, and cultural spaces, together with the dispositions, educational credentials, and educational upbringing activities that can influence other family members—constituting, beyond economic and social factors, the family’s cultural structure.

*Habitus*, in Bourdieu’s cultural capital theory, is the materialist path that agents acquire and create from the structure of practical experience in life. In short, habitus is a generative ability existing in the system of disposition tendencies as a skill, which is practiced, structurally constructed and shaped (Bourdieu and Wacquant, 1992, p. 122). *Field* can be defined as the objective relationship network or configuration existing among various positions (Bourdieu and Wacquant, 1992, p. 97).

In Bourdieu’s Theory of Practice, habitus and field are closely integrated. As the earliest form of cultural capital individuals encounter, family cultural capital can shape cognition and modes of thinking (Farkas, 2003), influence behavioral tendencies and aesthetic preferences (Hanquinet et al., 2014), and determine values and attitudes toward life (Holt, 1998). These cognitive, affective, and behavioral dispositions constitute components of an individual’s habitus, reflecting the impact of family cultural capital on habitus. Similarly, family cultural capital also defines the entry threshold of the field and influences the power structure within the field (Naidoo, 2004). That is, family cultural capital exerts a profound and complex influence on individual development through the shaping of habitus and its multi-faceted effects on the field. Therefore, this study proposed the following hypotheses:

*H1*: Family cultural capital has a significant impact on individual habitus.

*H2*: Family cultural capital has a significant impact on an individual’s position in their respective field.

#### 2.2.2 Influencing factors of college students’ academic achievements

The academic performance of college students is a quantifiable manifestation of their learning achievements at school. The most common way academic performance can show students’ mastery of course knowledge is in the summative form of points or grades (Fenollar et al., 2007). However, the academic performance of a student is the result of the complex interaction of various factors. For instance, factors such as a student’s learning habits, personality traits, teaching effectiveness, and gender differences can have an impact on the academic performance of college students (Arora and Singh, 2017). Since Coleman (1968) pointed out the significant correlation between family background and academic performance, some studies have revealed the educational inequality brought about by family cultural capital (Dumais and Ward, 2010; van de Werfhorst, 2010). Family cultural capital such as school participation, home-school interaction, and tutoring can have an impact on the relationship between families and schools, and thereby affect student’s educational experience (Lareau, 1987). Family cultural capital (Jin et al., 2024; Sullivan, 2001) influences educational achievements (DiMaggio, 1982; Tramonte and Willms, 2010), and educational equity. For college students, it also has an impact on academic performance (Dumais and Ward, 2010; Katsillis and Rubinson, 1990). Therefore, this study proposed the following hypothesis:

*H3*: Family cultural capital affects the academic achievements of college students.

#### 2.2.3 The mediating role of individual habitus

Family cultural capital can directly influence education through factors such as parental participation or educational investment, and it can also indirectly affect education by influencing students’ motivation. For instance, it can ultimately influence students’ academic performance through mediating factors such as students’ self-efficacy (Radulović et al., 2020), reading behavior (Myrberg and Rosén, 2008), and participation in extracurricular activities (Coulangeon, 2018). For the agent, habitus is a type of generative temperament and quality that emerges from individual practice. Habitus can be shaped by cultural capital and the field, and at the same time, it can also become a component of an individual’s cultural capital in the form of quality. The manifestation of habitus is a characteristic internalized as an individual’s behavioral habits and values, which can affect students’ performance during their schooling. These behavioral manifestations are related to teachers’ educational evaluations of students, and students’ knowledge acceptance during the educational process, both of which can have an impact on the final academic performance. Researchers have long noticed the important mediating role of habitus in Bourdieu’s Theory of Practice system, and have demonstrated this through empirical research (Edgerton et al., 2013; Gaddis, 2013). Therefore, this study proposed the following hypotheses:

*H4a*: Individual habitus has a direct and significant impact on the academic achievements of college students.

*H4b*: Individual habitus plays a mediating role between family cultural capital and the academic achievements of college students.

#### 2.2.4 The mediating role of the school field

As a complex field, school factors such as safety, interpersonal relationships, teaching and learning, facilities and infrastructure, and educational policies have an impact on students’ academic performance (Thapa et al., 2013). Consistent with Bourdieu (1986), cultural capital’s value is field-specific; thus, family cultural capital requires adaptation to the school field to yield benefits (Byun et al., 2012). In the process of students achieving excellent academic results, the school field performs the screening and differentiation of family cultural capital by continuously rewarding the family cultural capital with high consistency, and ultimately completes the beneficial distribution of power in the school field. In the process in which family cultural capital influences students’ academic performance, school field factors such as teachers’ educational behaviors (Wildhagen, 2009), teacher-student relationships (Raudenská, 2022), classmate relationships (Chiu and Chow, 2015), school management (Leithwood and Sun, 2018), and school facilities (Durán-Narucki, 2008) can play a mediating role. Therefore, this study proposed the following hypotheses:

*H5a*: The school environment has a direct and significant impact on college students’ academic achievement.

*H5b:* The school field plays a mediating role between family cultural capital and the academic achievement of college students.

#### 2.2.5 Sequential mediating effects of individual habitus and school fields

In Bourdieu’s Theory of Practice, human action—*or practice*—results from the dynamic interaction among capital, habitus, and field (Mouzelis, 2008). Bourdieu’s Theory of Practice is based on the concept of initiative. For the theory of practice, each individual is an agent, and they are active participants in the formation of the social world. Therefore, when we discuss the relationship between family cultural capital and school education, we must regard students as active actors (Clegg, 2011), and to a certain extent, students can influence and control their relationships and behaviors at school. In the field of higher education, students can formulate appropriate learning strategies and action plans through a detailed understanding of their experiences, backgrounds, and educational processes to help them achieve excellent academic results (James et al., 2015).

An individual’s habitus will determine how they utilize resources and interact with others in the school environment. For instance, students’ characteristics, positive and negative experiences, as well as cultural similarities, influence the establishment of relationships between students and teachers (Mallik, 2023). Students with proactive learning habits can actively participate in the teaching activities organized by teachers and are more likely to establish good relationships with classmates, thereby creating a positive micro-environment in the school field (David et al., 2024).

In *An Invitation to Reflexive Sociology*, Bourdieu and Wacquant (1992) emphasizes that habitus serves as the mediating link between individuals and fields. Individuals first develop stable dispositions through family cultural capital, then, guided by these dispositions, participate in field interactions, thereby adapting to or influencing the field’s rules. Habitus thus constitutes a prerequisite for the interplay between family cultural capital and the field; only by forming a congruent habitus can individuals access resources and gain recognition within the school field. This study posits the chained mediation pathway as “family cultural capital → habitus → field → academic achievement,” which aligns with the core logic of Bourdieu’s theory of practice. Therefore, this study proposed the following hypotheses:

*H6*: Individual habitus has a direct and significant impact on the school field.

*H7*: Individual habitus and the school field mediate family cultural capital and college students’ academic achievement.

**FIGURE 1:**
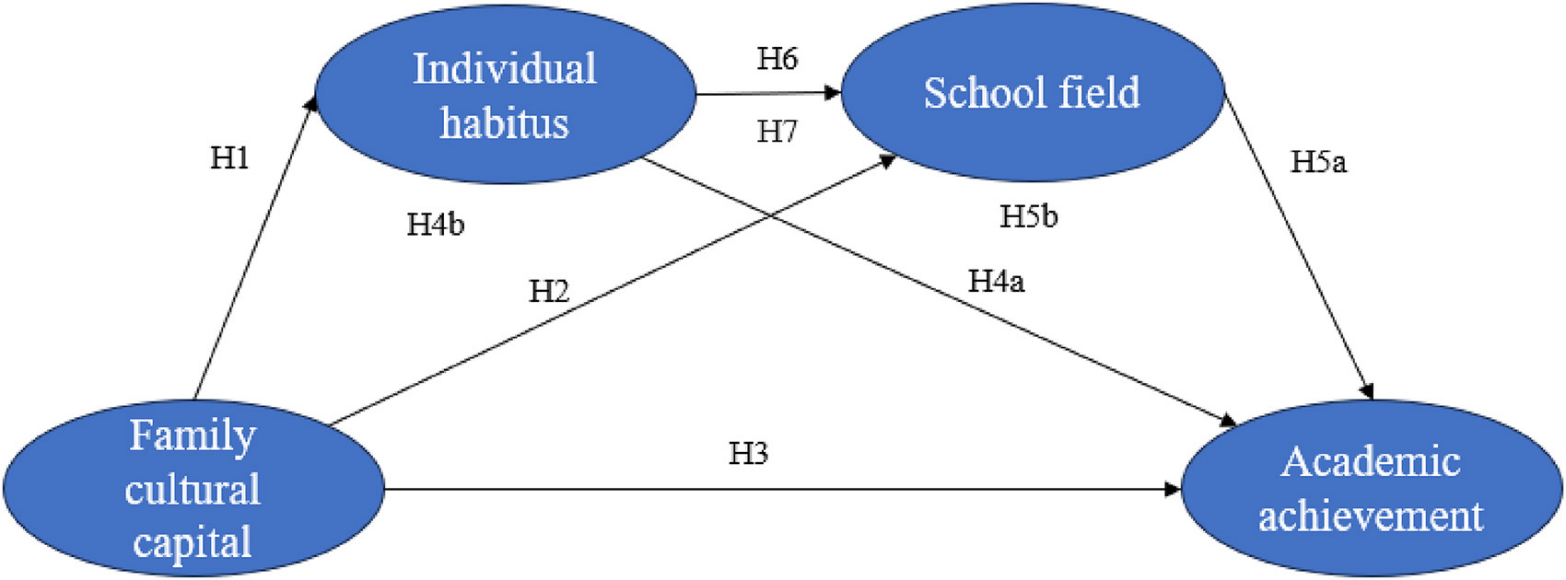
Conceptual model.

Figure 1 shows the conceptual model of this study’s hypothesis development.

## 3 Materials and methods

### 3.1 Measurement

The questionnaire in this study was scored using a five-point Likert scale (1 indicated *strong disagreement*, and 5 indicated *strong agreement*). The measurement of family cultural capital in the questionnaire items adopted the scale items of Tan (2017), Tan et al. (2019), and Yuan et al. (2016), covering three dimensions: Family relationship, family support, and family interaction respectively. The measurement of individual habitus-taking was derived from the scale items of Tan and Liu (2022) and Gaddis (2013), including three dimensions: Personal expectations, learning engagement, and learning strategies. The measurement of the school field was adapted from Greenwald et al. (1996), Hampden-Thompson and Galindo (2017), and Sengul et al. (2019), covering two aspects: the teacher-student relationship and the classmate relationship. The measurement of college students’ academic performance adopted the self-reporting model commonly used by previous researchers, including measurement items such as academic performance, competition awards, and scholarships. This study’s questionnaire also contained demographic data, such as gender, age, year of study, type of school, and major. In addition, as some of the questionnaire items were in English, the research team also invited native English speakers to translate the relevant items to improve the questionnaire’s content validity.

### 3.2 Data collection

This study took China as the research site. China currently has 3,074 higher education institutions, with a total enrollment of 47.63 million students across all forms of higher education (Ministry of Education, 2024). In the specific implementation process of the research, due to limitations in time and funding, random sampling was not employed as the only sampling procedure. The combination of convenience sampling and stratified random sampling was deemed a reasonable method to obtain a large amount of data in this study (Hedt and Pagano, 2011). Data collection was conducted with traditional paper questionnaires.

Through the collaborative network of the researcher’s institution, contact was made with 12 higher education institutions (9 four-year undergraduate universities and 3 vocational colleges) across five provinces/municipalities–Beijing, Zhejiang, Jiangsu, Henan, and Inner Mongolia. Within each institution, stratified sampling was conducted based on academic discipline categories, year of study, and gender to recruit volunteer participants, ensuring sample diversity. The main study employed a postal survey method, mailing paper questionnaires to volunteer participants selected through convenience sampling from the target institutions. Following the principles of stratified random sampling, the distribution and collection of paper questionnaires were conducted upon obtaining informed consent from the participants and ensuring compliance with academic ethics regulations.

A pilot test was conducted in October 2024, and a total of 128 valid questionnaires were collected. The pilot test was carried out at Hangzhou Normal University and Tourism College of Zhejiang, where the researchers are affiliated. A prior sample size calculation was carried out to estimate the number of participants required by the model. After the preliminary analysis of the pilot test data, the standardized Cronbach’s α coefficient was 0.932, which was higher than 0.8, indicating that the reliability of the scale items was high.

Subsequently, the main study questionnaire was conducted from November 2024 to February 2025. A total of 3,000 questionnaires were distributed, and 2,929 valid questionnaires were retrieved, with an effective return rate of 97.63%. The demographic information of the valid questionnaire respondents is shown in Table 1.

**TABLE 1:** Demographic profile of the questionnaire participants.

Variable	Category	*n*	%
Gender	Male	1,257	42.92
	Female	1,672	57.08
Year of study	Freshman	703	24.00
	Sophomore	690	23.56
	Junior	926	31.61
	Senior	610	20.83
Major	Science and technology	493	16.83
	Literature and history	782	26.70
	Economics and management	809	27.62
	Agriculture, forestry, and Medicine	105	3.58
	Others	740	25.26
School type	4-Year undergraduate university	2,038	69.58
	Higher vocational college	891	30.42

*N* = 2,929.

## 4 Results

### 4.1 Measurement properties

Data analysis and model measurement were conducted using SPSS 25.0 and AMOS 24.0 software. First, skewness and kurtosis tests were conducted on the sample data to evaluate the normality of the collected data. In this study, the skewness of each item was between –0.698 and 0.28, and the kurtosis of each item was between –0.157 and 0.89, indicating that the data exhibited a normal distribution (Joanes and Gill, 1998). Confirmatory factor analysis was conducted for 9 factors and 40 analysis items. The effective sample size was 2,929, which was 10 times more than the number of analysis items, so the sample size was appropriate.

After validity analysis, the common degree values corresponding to the questionnaire items in this study were all higher than 0.4, indicating that the information of the questionnaire items could be effectively extracted. The validity was verified using the KMO and Bartlett tests. The KMO value was 0.935, which was > 0.8. The KMO test assesses sampling adequacy for factor analysis, providing strong support for the construct validity of the questionnaire items. In addition, the variance explanation rate values of the 9 factors were 14.107, 7.559, 7.496, 7.284, 6.578, 6.477, 6.197, 5.765, and 5.371%, respectively. The cumulative variance explanation rate after rotation was 66.83%, > 50%. This meant that the amount of information from the questionnaire item could be effectively extracted.

Subsequently, confirmatory factor analysis was conducted to examine whether the correspondence between the measurement factors and the measurement items was consistent with the researchers’ predictions, enhancing the validity and reliability of the data. After analysis, the model fitting indicators were as follows: GFI = 0.914, RMSEA = 0.049, RMR = 0.026, CFI = 0.922, NFI = 0.912, NNFI = 0.914. Among them, χ^2^/*df* = 7.908. Because the chi-square value expands with the increase of the sample size, when the sample size is large, the representativeness of the chi-square value/degree of freedom index is weak (Bearden et al., 1982; Powell and Schafer, 2001). Therefore, all the indicators of the data in this study were within an acceptable range. According to the factor loading coefficient values, there was a strong correlation between the latent variables and the bound variables in this study. Confirmatory factor analysis (CFA) was conducted for 9 factors and 40 analysis items. The AVE values corresponding to the 9 factors were all > 0.5, and the CR values were higher than 0.7, indicating that the data had good convergent validity (see Table 2).

**TABLE 2:** Confirmatory factor analysis results.

Construct/items	Factor loading	Cronbach’s α	AVE	CR
Family relationship		0.871	0.630	0.872
Family relations are harmonious	0.793			
Get along well with my father	0.812			
Get along well with my mother	0.795			
The relationship between parents is harmonious	0.774			
Family support		0.829	0.619	0.830
Support participation in extracurricular activities	0.778			
Support participation in sports activities	0.79			
Support participation in art activities	0.792			
Family interaction		0.840	0.569	0.841
Exchange school matters	0.766			
Communicate matters related to friends	0.775			
Exchange worries or troubles	0.743			
Exchange about life development	0.735			
Personal expectation		0.854	0.595	0.854
Higher academic qualifications	0.739			
Higher social status	0.787			
Higher economic status	0.789			
Higher personal achievements	0.769			
Learning engagement		0.911	0.533	0.911
When studying, I am energetic	0.758			
Even if my learning doesn’t go smoothly, I won’t be discouraged and I can persist	0.737			
When studying, I am full of energy	0.758			
Learning is challenging	0.711			
Be passionate about learning	0.751			
The learning purpose is clear and very meaningful	0.742			
When studying, time passes quickly	0.709			
When studying, I focus on it	0.713			
When studying, I feel happy	0.691			
Learning strategy		0.809	0.516	0.810
In the process of learning, I will consciously think, organize and process the learning content	0.692			
In the learning process, goals will be set and self-monitoring will be carried out	0.733			
Have an autonomous consciousness in learning	0.753			
I can feel the value of learning	0.693			
Teacher-student relationship		0.885	0.660	0.886
Communicate emotional confusion with the teacher	0.775			
Communicate with teachers about academic studies	0.842			
Communicate with the teacher about daily life	0.819			
Communicate with the teacher about life planning	0.812			
Classmate relationship		0.809	0.514	0.809
I Have good friends at school	0.703			
The atmosphere in the class is very good	0.704			
The atmosphere in the dormitory is very good	0.707			
My interpersonal relationships at school are very good	0.754			
Academic achievement		0.798	0.507	0.801
My comprehensive academic performance ranks high in the class	0.544			
I participate in professional and subject-related competition activities	0.799			
I participate in cultural and sports competitions and activities	0.796			
I obtain a scholarship	0.68			

Table 3 presents the discriminant validity analysis results for each construct. The square roots of the average variance extracted (AVE) for all factors exceeded the absolute values of their correlation coefficients with other factors, demonstrating adequate discriminant validity.

**TABLE 3:** Discriminant validity: Pearson correlation with the AVE square root value.

Dimension	Family relationship	Family support	Family interaction	Personal expectation	Learning engagement	Learning strategy	Teacher-student relationship	Classmate relationship	Academic achievement
Family relationship	**0.794**								
Family support	0.521	**0.787**							
Family interaction	0.514	0.563	**0.755**						
Personal expectation	0.334	0.358	0.331	**0.771**					
Learning engagement	0.324	0.368	0.48	0.423	**0.73**				
Learning strategy	0.324	0.388	0.43	0.414	0.636	**0.718**			
Teacher-student relationship	0.333	0.325	0.421	0.247	0.469	0.399	**0.812**		
Classmate relationship	0.419	0.384	0.411	0.393	0.449	0.425	0.542	**0.717**	
Academic achievement	0.108	0.175	0.252	0.168	0.382	0.357	0.376	0.222	**0.712**

The bold numbers on the diagonal lines represent the square root values of AVE.

### 4.2 Assessment of the structural model

**FIGURE 2:**
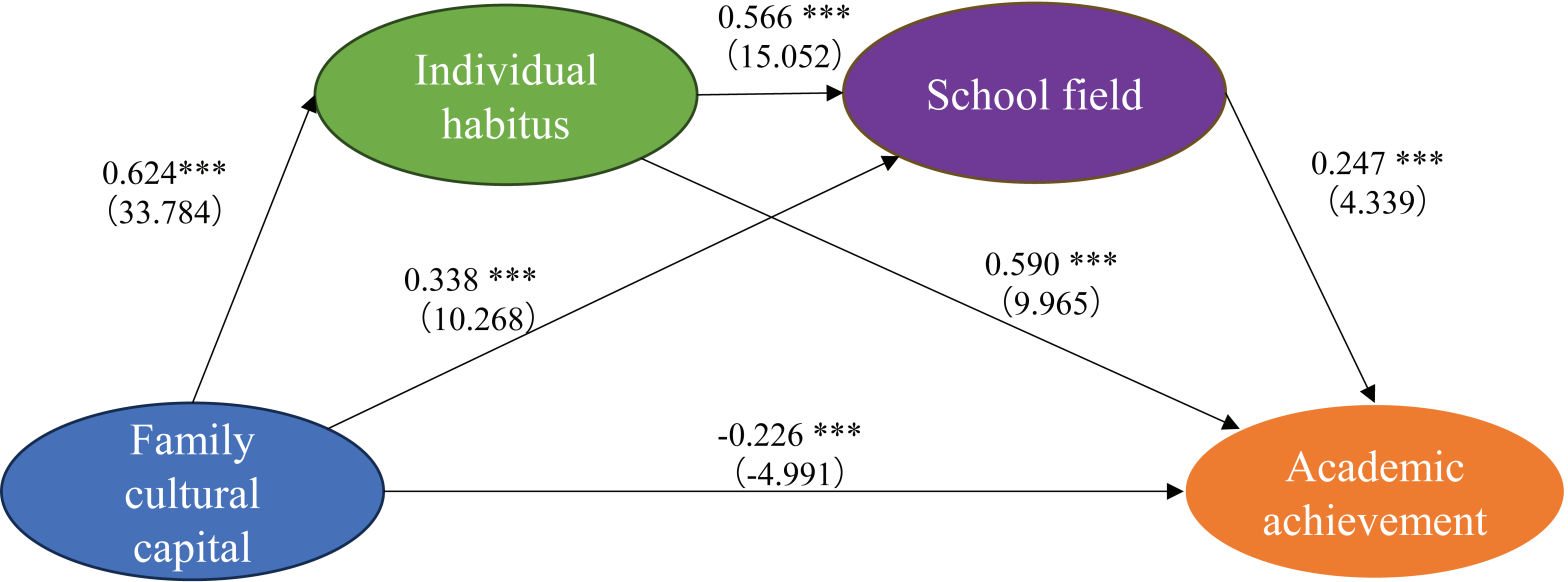
AMOS output results of the proposed model. The path coefficients for family cultural capital and academic performance (β = −0.226, *p* < 0.001), family cultural capital and habitus (β = 0.624, *p* < 0.001), family cultural capital and field (β = 0.338, *p* < 0.001), habitus and academic performance (β = 0.590) (*p* < 0.001), field and academic performance (β = 0.247, *p* < 0.001), and habitus and field (β = 0.566, *p* < 0.001), presented significant results. Therefore, H1, H2, H3, H4a, H5a, and H6 in the research hypotheses were supported.

The structural equation model was modeled in AMOS to obtain the model fitting results. Among them, χ^2^/*df* = 8.38, GFI = 0.898, RMSEA = 0.050, RMR = 0.041, CFI = 0.910, NFI = 0.899. These values are within a reasonable range. Therefore, the goodness of fit is relatively high in this model (see Figure 2 and Table 4).

**TABLE 4:** Structural model assessment and hypothesis test results.

Hypothesis	Path	Standardized coefficient	*T*-value	Result
H1	Family cultural capital → habitus	0.624	33.784***	Supported
H2	Family cultural capital → field	0.338	10.268***	Supported
H3	Family cultural capital → academic achievement	−0.226	−4.991***	Supported
H4a	Habitus → academic achievement	0.590	9.965***	Supported
H5a	Field → academic achievement	0.247	4.339***	Supported
H6	Habitus → field	0.566	15.052***	Supported

***p < 0.001.

### 4.3 Assessment of mediation effects

The estimators of mediation analysis are highly sensitive to situations that deviate from the normality assumption. To test the reliability of the mediation effect, the bootstrapping method was used (Alfons et al., 2022). As many as 5,000 bootstrap replications are generally sufficient to stabilize the confidence intervals of mediation effects. Bootstrapping tests were conducted on sensitive data, such as outliers, including heavy tails or the skewness of the observed distribution that may have threatened the proposed mediating mechanism, In Table 5, the total effect of family cultural capital on college students’ academic performance [95% CI (0.252, 0.376)], and the direct influence of family cultural capital on college students’ academic performance [95% CI [–0.338, –0.123)] are presented. The indirect influence of family cultural capital on college students’ academic performance through individual habitus (95% CI [0.284, 0.470]), and the indirect influence of family cultural capital on college students’ academic performance through the school field (95% CI [0.03.8, 0.146]) are also shown. These results indicate that family cultural capital has an indirect influence on college students’ academic performance successively through individual habitus and the school field [95% CI (0.044, 0.140)], and their confidence intervals do not include zero values. Therefore, a significant mediating effect was revealed.

**TABLE 5:** Path mediation results.

	Estimation	Bootstrapping 5,000		*P*-value
Path		Percentile 95% CI		
		Lower	Upper	
**Total effect**				
	0.313	0.252	0.376	0.000
**Direct effect**				
	−0.226	−0.338	−0.123	0.000
**Indirect effect**				
H4b: Family cultural capital → Habitus → Academic achievement	0.368	0.284	0.470	0.000
H5b: Family cultural capital → Field → Academic achievement	0.084	0.038	0.146	0.000
H7: Family cultural capital → Habitus → Field → Academic achievement	0.087	0.044	0.140	0.000

## 5 Discussion

### 5.1 Conclusion

This study investigated the relationship among family cultural capital, individual habitus, school field, and academic performance among Chinese college students. The findings support the influence of family cultural capital on college students’ academic performance mentioned in previous studies (Dumais and Ward, 2010), as well as the mediating role played by habitus and field in this process (Chiu and Chow, 2015; Edgerton et al., 2013; Gaddis, 2013; Raudenská, 2022). In addition, the significant chain mediating effect among family cultural capital, habitus, school field, and academic performance also bolsters the research applicability of Bourdieu’s Theory of Practice.

Three findings warrant further discussion. First, the research results showed that the direct impact of family cultural capital on the academic performance of Chinese college students is significantly negatively correlated. This phenomenon, which is inconsistent with the original assumption of Bourdieu’s theory of cultural capital, confirms that not all cultural capital can directly have a favorable impact on students’ academic performance (Tan et al., 2019). That is, the value of cultural capital in studies focusing on academic performance is not that salient (Byun et al., 2012; Yamamoto and Brinton, 2010). This study utilized the data of Chinese college students to illustrate that in higher education, the direct influence of family cultural capital on academic performance has certain limitations. Especially in Chinese society, family cultural capital is deeply influenced by Confucian culture (Hu, 2023). An environment with high family cultural capital may lead to overly high expectations from parents. It is possible these high parental demands can make some Chinese college students develop a rebellious mentality, which could negatively affect their academic performance.

Second, the results showed that individual habitus and the school field have a significant positive impact on Chinese college students’ academic performance. This study took personal expectations, learning engagement, and learning strategies as latent variables for measuring individual habitus, confirming the important mediating role of self-factors between family cultural capital and academic performance. This result supports the mediating role of habitus factors such as achievement goals and self-efficacy on academic performance (Fenollar et al., 2007). This suggests that a significant influence of Chinese college students’ initiative on their academic performance can be a factor. Similarly, this study takes the teacher-student relationship and the classmate relationship as the latent variables for measuring the school field, confirming that the relationship structure in the school, such as the educational and teaching methods, can have an impact on Chinese college students’ academic performance. In other words, the mediating effect of the school field suggests that cultural capital needs to be linked to the field in which it functions to form value (Davies, 2024).

Third, this study found that, among the sampled Chinese college students, family cultural capital has an indirect positive impact on academic performance through the sequential path of individual habitus and the school field. This indicates that for advantageous family cultural capital to be transformed into excellent academic achievements, it must undergo capital transformation and adaptation to align it with the requirements of school education. This finding supports Bourdieu’s discussion on the original meaning of cultural capital (Bourdieu and Passeron, 1977). This chain-like mediating effect reveals the distinctive features between cultural capital and economic capital. Unlike economic capital, cultural capital cannot be directly passed on to the next generation. It must go through a long period of accumulation, transformation, and field adaptation to gain benefits in a specific field.

### 5.2 Implications for theory

The findings of this study not only enrich the tenets of Bourdieu’s Theory of Practice, but also promote the transformation of the application of cultural capital theory from *resource analysis* to *relationship analysis* and *process analysis*, providing a referable theoretical integration paradigm and methodological innovation path for subsequent research. The theoretical contributions are reflected in three aspects: an innovative application of Bourdieu’s Theory of Practice system, an expansion of the theoretical framework, and a novel interpretation of the core issues of educational sociology.

First, based on Bourdieu’s Theory of Practice, this study constructed an integrated analysis framework of *capital–habitus–field* interaction, overcoming the limitation of a single-factor analysis strategy. This framework incorporates the core concepts in Bourdieu’s Theory of Practice into a unified analytical tool. Through empirical testing, this study examined the chain mediating effect of family cultural capital on academic performance among Chinese college students in a multi-factor method, moving beyond the single-factor analysis of cultural capital in traditional research. This tripartite theoretical framework not only retains the core logic in Bourdieu’s theory that capital relies on the field to exert its value (Bourdieu, 1986), but also extends the mechanism of interdependence and entanglement among the three constructs (capital–habitus–field) in the Theory of Practice system (Breiger, 2000). This novel capital–habitus–field framework provides empirical support for understanding the potential mechanism by which cultural capital is internalized through individual habitus and adapted to the field to be transformed into academic advantages. In sum, this tripartite interaction framework appears to provide a more comprehensive theoretical analysis tool for research in educational sociology in the Chinese context.

Second, in contrast to the prevailing assumptions about cultural capital theory, this study reframes it by highlighting field specificity and localized context. This study’s findings suggest that the direct impact of family cultural capital on academic performance among Chinese colleges students is significantly negatively correlated, challenging the traditional perception that cultural capital necessarily promotes educational achievements. This reveals that in China—due to the differences in educational fields, examination models, and social mechanisms—the types of cultural capital and the operation mechanisms of the fields that can affect academic achievement are different from those outside China (e.g., North America and Europe) (Davies, 2024). In addition, in the Chinese context, this significant negative direct effect may stem from the rebellious psychology of students caused by overly high expectations from families under Confucian culture, as well as the low adaptability of the East Asian examination system to refined cultural capital (e.g., museum visits, attending concerts, and theatrical performances), suggesting that the value of cultural capital is determined by the specific field. This interpretation aligns with Bourdieu’s Theory of Practice and provides preliminary evidence for its explanatory power in the socio-cultural paradigm of Chinese higher education.

Third, this study reveals the micro-interaction mechanism between habitus and field, expanding the theoretical implications of educational equity research in China. The mediating role of individual habitus factors such as learning strategies, learning engagement, and self-expectations, as well as school field factors such as teacher-student relationships and classmate interactions in the transformation of cultural capital was found in this study. The discovery of this mechanism is similar to Randall Collins’ micro-interactive ritual theory and represents a theoretical refinement to Bourdieu’s cultural capital theory (Davies and Rizk, 2018). Specifically, habitus serves as a bridge between objective structures and subjective actions, both of which are not only shaped by family cultural capital but also actively influence individuals’ behavioral strategies in the school field. This research conclusion contradicts the limitations of the traditional view that family resources determine the acquisition of educational outcomes. It also deepens the connection between family background and academic achievements into a dynamic process analysis of capital transformation → habitus generation → field interaction, providing a new perspective for understanding the complex causes of educational inequality in China.

### 5.3 Implications for education practice

This study provides multi-dimensional application value for Chinese higher education policy-making, educational practice, and family education by revealing the dynamic relationship among family cultural capital, habitus, field, and academic performance. This dynamic relationship is reflected in the following three implications for education practice.

First, in the formulation of educational policies, this application value promotes a reformulation of family cultural capital and the school field. In the process of formulating educational policies in the Chinese government, the home–school collaboration mechanism can be optimized. In response to the possible negative impact on academic performance caused by expectation mismatch in families with high cultural capital, families can be guided through parent education programs to transform cultural capital into supportive habitus cultivation rather than directly transmitting refined cultural capital, such as artistic cultivation and elite values (Kim, 2022). During the policy-making process, in light of the disadvantages of families with low cultural capital, habit-empowering policies such as learning strategy training and psychological efficacy improvement can be formulated. The strong mediating effect of habitus shown in the results of this study can be utilized to make up for the disadvantages of family resources by promoting families and students to adapt to the rules of the school environment through positive individual habitus.

Second, in the practice of higher education in China, a dual intervention system of habitus cultivation and field optimization should be constructed. In the formulation of teaching strategies in schools, emphasis should be placed on the improvement of habitus (Wong and Liao, 2022). To achieve this, teachers can provide differentiated guidance by evaluating students’ learning strategies, goal expectations, and other habitual classroom indicators. For instance, students with high family cultural capital could be guided by their teachers to transform family advantages into innovative abilities. On the other hand, teachers could strengthen basic learning strategies to directly improve academic performance among students with low family cultural capital. Chinese universities and colleges should also improve the micro-social network on campus. By building an equal teacher–student interaction and peer support network, the relationship structure of the school field could be optimized. This optimization would reduce the exclusion caused by the mismatch of family cultural capital, and enable students from different backgrounds to accumulate beneficial habitus through positive interactions with their teachers and peers on campus.

Third, in family education in China, emphasis should be placed on shifting from the transmission of capital to the empowerment of habitus. To start, Chinese parents need to adjust their role positioning in the family. That is, families with high cultural capital should try to avoid cultural coercion. They can cultivate children’s active learning habitus through democratic discussions, independent decision-making, companionship, mentoring, and guidance. On the contrary, families with low cultural capital can focus on non-material investment and take advantage of the shaping effect of habitus on the field to enhance their children’s adaptability at school. Finally, the initiative of individual students should also be cultivated. Personalized guidance can help students identify their strengths, formulate action strategies suitable for the school field, and indirectly transform family cultural capital into academic advantages.

In conclusion, by recognizing and harnessing the chain-like intermediary nexus between habitus and field—regardless of the level of family cultural capital—students’ adaptability to the school field can be enhanced by cultivating positive habitus, and the impact of family cultural capital on educational inequity can be mitigated in Chinese society.

### 5.4 Limitations

Through empirical analysis, this study revealed the concatenating mediating effect of individual habitus and the school field between family cultural capital and Chinese college students’ academic performance. The research results provide a fresh lens to see beyond the traditional limitations of family cultural capital theory and can contribute to a more equitable development of Chinese education in multiple aspects, creating a fairer and more effective educational field for students.

However, our study also has certain limitations. First, due to the complexity of family cultural capital theory and challenges in its measurability, the theoretical framework and variable measurement were simplified, which may have introduced some measurement error. Second, the study employed cross-sectional data, which precludes capturing the dynamic evolution of family cultural capital, habitus, and field over time. Third, owing to constraints in sample collection, the research was conducted in a selection of Chinese higher education institutions; therefore, caution should be exercised when generalizing the findings to universities in other countries and regions or to other stages of education.

In future research, several directions merit further exploration to deepen the understanding of the relationship between family cultural capital and student academic performance: (1) Theoretical integration should be enhanced to bridge existing frameworks and offer more comprehensive explanatory power; (2) More dynamic and diverse samples are needed to reflect varying educational contexts and social backgrounds; (3) Key variables related to cultural capital and academic achievement require further refinement and operationalization; (4) Greater emphasis should be placed on contextualizing the transformation of cultural capital within the specific trajectory of China’s educational reform; and (5) Incorporating formative evaluations through qualitative research methodologies—such as interviews, focus groups, and case studies—can yield deeper insights into the nuanced mechanisms through which family cultural capital affects student outcomes and academic performance.

## Data Availability

The raw data supporting the conclusions of this article will be made available by the authors, without undue reservation.
